# Variation in Extracellular Polymeric Substances from *Enterobacter* sp. and Their Pb^2+^ Adsorption Behaviors

**DOI:** 10.1021/acsomega.1c00185

**Published:** 2021-04-01

**Authors:** Yi Li, Meifen Xin, Dongyu Xie, Shirui Fan, Jiangming Ma, Kehui Liu, Fangming Yu

**Affiliations:** † Key Laboratory of Ecology of Rare and Endangered Species and Environmental Protection (Guangxi Normal University), Ministry of Education, 541004 Guilin, China; ‡ College of Environment and Resources, Guangxi Normal University, 541004 Guilin, China; § College of Life Science, Guangxi Normal University, 541004 Guilin, China; ∥ Innovation Institute of Sustainable Development, Guangxi Normal University, 541004 Guilin, China

## Abstract

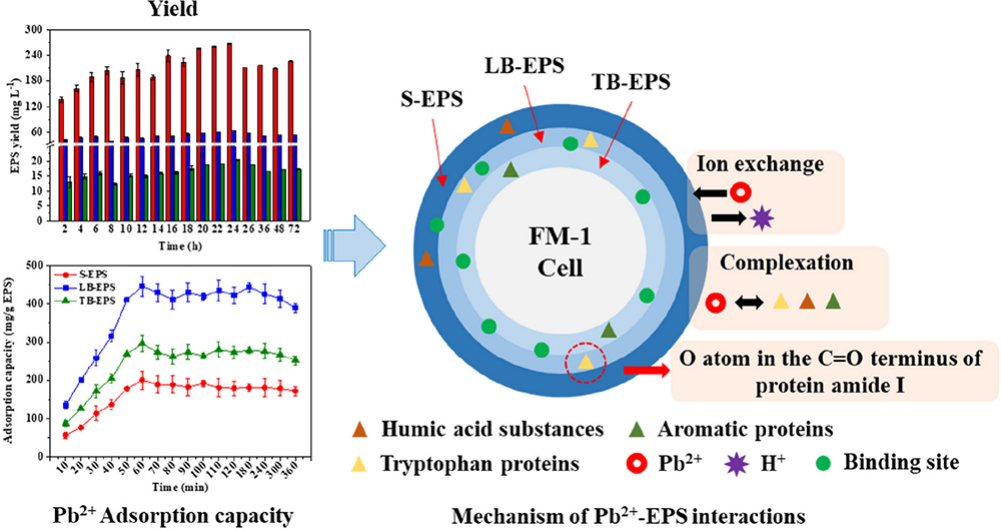

The objective of this study was to investigate the effects of the cultivation time, temperature, and pH value on the yield and composition of extracellular polymeric substances (EPS) from *Enterobacter* sp. FM-1 (FM-1) and to analyze the Pb^2+^ adsorption behavior of soluble EPS (S-EPS), loosely bound EPS (LB-EPS), and tightly bound EPS (TB-EPS). Maximum EPS production was obtained when the cultivation time, temperature, and pH value were 24 h, 30 °C, and 8.0, respectively. The main components of EPS were proteins, polysaccharides, and nucleic acids, but the different EPS types contained different proportions and specific components. The Pb^2+^ adsorption capacity of LB-EPS was 2.23 and 1.50 times higher than that of S-EPS and TB-EPS, respectively. After Pb^2+^ adsorption by LB-EPS, the pH value of the reaction system decreased to the lowest of 5.23, which indicated that LB-EPS contained more functional groups that could release H^+^, which will help to better adsorb Pb^2+^ through ion exchange. The three-dimensional excitation–emission matrix fluorescence spectroscopy (3D-EEM) analysis showed that the fluorescence intensity of tryptophan-containing substances decreased by 85.5% after Pb^2+^ adsorption by LB-EPS, which indicated the complexation of tryptophan-containing substances with Pb^2+^. Fourier transform infrared spectroscopy (FT-IR) and X-ray photoelectron spectroscopy (XPS) O spectra indicated that the C=O peak from protein amide I of tryptophan-containing substances in LB-EPS was mainly responsible for the complexation of Pb^2+^. After the adsorption of Pb^2+^, the proportion of the C=O peak in LB-EPS increased by 33.89%, indicating that the complexation of LB-EPS with Pb^2+^ was mainly attributed to the O atom in the C=O terminus of protein amide I.

## 1 Introduction

Lead (Pb) is a highly toxic metal that causes environmental contamination worldwide and is also a nonessential metal for humans.^1^ In particular, Pb poisoning is common among children and will increase the risk of neurotoxic disorders and lead to mental retardation.^2^ Moreover, Pb is toxic to microorganisms by damaging DNA, inhibiting enzyme activity, and disrupting the permeability of the cell membrane.^3^ Guangxi Province in China has rich reserves of Pb–zinc (Zn) mines, and exploitation activity will cause irreversible Pb pollution to the surrounding soil and aquatic ecosystems.^4^ Recently, Pb remediation techniques, including ion exchange, chemical immobilization, membrane filtration, phytoremediation, have been used and evaluated for their Pb removal efficiency.^5−8^ In contrast to physicochemical methods, which might exhibit significant demerits and restrict their widespread application, adsorption based on microorganisms has developed rapidly and has wide application prospects in the remediation of heavy metal (HM) pollution.^3,9^

Generally, HM bioaccumulation in microorganisms arises from metabolism-dependent intracellular accumulation and metabolism-independent extracellular adsorption (surface interaction between HM ions and cell surface components).^10−12^ Extracellular polymeric substances (EPS), which are metabolites secreted by microorganisms into the extracellular environment during the process of growth and metabolism, play an important role in the extracellular adsorption of HMs by microorganisms.^5,13^ EPS are a complex mixture of macromolecular substances surrounding the cells of microorganisms that mainly comprise polysaccharides, humic-like substances, proteins, nucleic acids, lipids, and other molecules.^14,15^ The forms of EPS are divided into soluble EPS (S-EPS) and bound EPS (B-EPS), among which B-EPS are divided into tightly bound EPS (TB-EPS) and loosely bound EPS (LB-EPS) according to whether they are closely wrapped around the outside of the cells of microorganisms.^16^ According to previous studies, the types and components of EPS secreted by microorganisms may vary substantially under different environmental conditions.^17^ In addition, changes in the composition, functional group content, and surface microstructure of EPS will affect the adsorption of HMs by EPS; among these factors, the composition of EPS determines the type and content of functional groups, thereby determining the HM binding sites and amount of HM adsorbed.^18^ Cai et al.^19^ investigated the LB-EPS and TB-EPS produced by the strain *Aeromonas veronii* N8 upon Zn^2+^ stimulation. These two types of EPS contained different proportions of polysaccharides and proteins, and thus, their adsorption mechanisms differed. Similarly, Hong et al.^20^ indicated that LB-EPS produced by a *Brevibacillus agri* strain presented an excellent Pb^2+^ adsorption capacity due to their alveolar shape and large specific surface area. Based on these studies, microorganisms can adsorb HMs by secreting EPS. However, due to the diversity of microorganisms, large discrepancies exist among the characteristics, composition, and structure of EPS secreted by microorganisms.

Metal adsorption by EPS is considered a self-protection strategy to defend the cells of microorganisms against toxic substances.^21^ The microbial EPS matrix contains abundant functional groups that are negatively charged under neutral conditions, such as N–H, C=O, and C–H. Thus, EPS not only have an ion exchange capability but also interact with HM ions.^22^ Because the metal adsorption behavior depends to a large extent on the properties of the functional groups, an understanding of the metal–EPS binding mechanism might help to explain the metal–cell interaction. For instance, Shen et al.^18^ found that EPS produced by *Synechocystis* sp. PCC6803 mainly complexed with Cd^2+^ through O–H and N–H groups. Xu et al.^23^ revealed that the EPS secreted by the strain *Pseudomonas putida* X4 reacted with Cd^2+^ through complexation to reduce the toxicity of Cd^2+^. However, the EPS from different types of microbes are quite different, and substantial differences exist in the biosorption processes of heavy metals, the interaction mechanism with heavy metals, and the functional groups that play a vital role in the adsorption process; all of these factors require further study. Therefore, an in-depth analysis of the behaviors of EPS and their metal binding mechanism will promote a better understanding of metal bioadsorption processes.

In the current study, *Enterobacter* sp. FM-1 (FM-1) (GenBank accession number MF664375), which was isolated from HM-contaminated soil in our previous study,^24^ was used as the target microbial species. The objectives of this study were (a) to investigate the changes in the yield and composition of EPS produced by FM-1 under different cultivation conditions and evaluate their adsorption capacities and the contributions of different EPS fractions to Pb^2+^ adsorption, (b) to characterize the variation in EPS produced by FM-1, and (c) to explore the detailed information mechanism of Pb^2+^ adsorption by EPS produced by FM-1. The results of this study will help to provide new insights into the mechanism underlying the interaction between different EPS fractions and Pb^2+^.

## 2 Results

### 2.1 Evaluation of the Pb^2+^ Tolerance of FM-1

**Figure 1:**
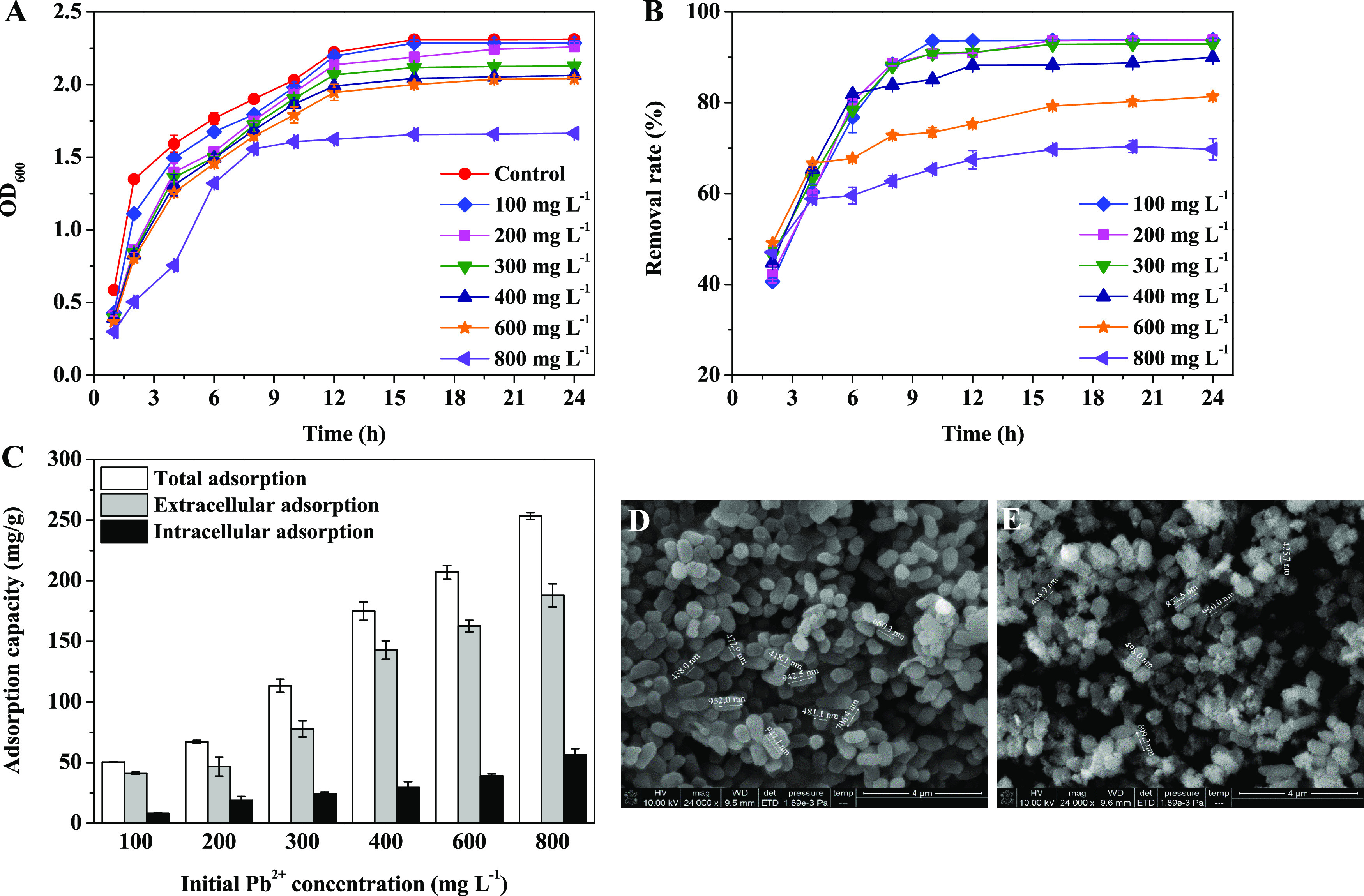
*Enterobacter* sp. FM-1 growth during 24 h of incubation in different concentrations of Pb^2+^ (a), Pb^2+^ removal rate (b), adsorption capacity of *Enterobacter* sp. FM-1 extracellular and intracellular (c), and SEM analyses of *Enterobacter* sp. FM-1 cultivated with (d) and without (e) Pb^2+^. The error bars represent the SD (*n* = 3).

The growth of FM-1 in an LB culture medium containing different concentrations of Pb^2+^ over 24 h, the Pb^2+^ removal efficiency, and the extracellular and intracellular adsorption capacity of FM-1 over 24 h are presented in Figure 1a–c. FM-1 was able to grow in the LB medium with a relatively high Pb^2+^ concentration (≤600 mg L^–1^). The growth of FM-1 was inhibited when the Pb^2+^ concentration reached 800 mg L^–1^, and the OD_600_ value was considerably lower than that of the control. Moreover, the Pb^2+^ removal efficiency presented in Figure 1b indicated that the removal efficiency of FM-1 increased dramatically during the first 2 to 12 h and then became stable after 12 h. Different initial Pb^2+^ concentrations exerted clear effects on the FM-1 removal efficiency. When the initial Pb^2+^ concentration was 100 mg L^–1^, the removal efficiency reached a maximum of 93.87% in 24 h; however, when the initial Pb^2+^ concentration was 800 mg L^–1^, the removal efficiency decreased to a minimum of 70.33%. As shown in Figure 1c, the extracellular and intracellular adsorption capacity of FM-1 increased over 24 h with increasing Pb^2+^ concentrations, and extracellular adsorption was the main adsorption mode.

SEM observations showed a smooth surface of FM-1 grown in the culture medium without Pb^2+^, and the outline was clear. The individual bacteria were clearly capsule-shaped, with lengths and widths ranging from 706.4 to 952.0 nm and 418.1 to 481.1 nm, respectively (Figure 1d). However, after Pb^2+^ (400 mg L^–1^) adsorption, the length and width ranged from 699.2 to 950.0 nm and 425.7 to 498.0 nm, respectively. Moreover, flocculated sediments around the cells were observed, which were likely attributed to the EPS secreted by the bacterial cells (Figure 1e).

### 2.2 Effects of Culture Time, Temperature, and pH on the Yield and Composition of Different EPS Fractions

**Figure 2:**
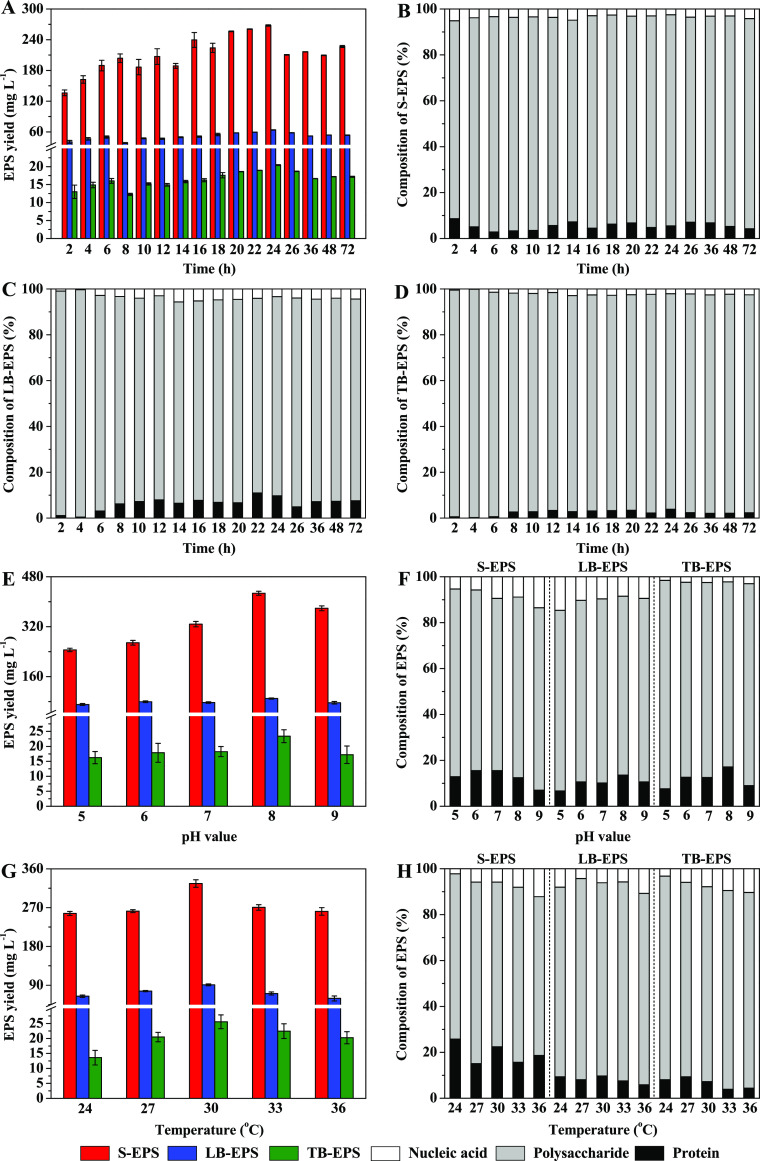
EPS yield under different cultivation times (a). Composition of S-EPS (b), LB-EPS (c), and TB-EPS (d) under different cultivation times. EPS yield (e) and composition (f) under different pH values. EPS yield (g) and composition (h) under different cultivation temperatures.

As shown in Figure 2a, during the first 24 h of cultivation, the yields of S-EPS, LB-EPS, and TB-EPS increased with increasing cultivation time and reached maxima of 267.78, 63.85, and 20.43 mg L^–1^, respectively, at 24 h. After 24 h, the yields of the three types of EPS decreased slightly. During the cultivation process, the proportion of S-EPS was the highest at approximately 76.06%, and the proportions of LB-EPS and TB-EPS were approximately 18.14 and 5.80%, respectively. The composition of different EPS fractions during the cultivation time is presented in Figure 2b–d. For S-EPS, LB-EPS, and TB-EPS, the polysaccharide portion was higher than the protein portion and nucleic acid portion, and the polysaccharide portion accounted for 91.02, 89.97, and 95.61% of the yield, respectively. The protein portion of S-EPS first increased and then decreased during the 24 h of cultivation. The protein portions of LB-EPS and TB-EPS increased slightly by 10.98 and 3.88%, respectively, at 24 h.

The effects of the culture pH on the yield and composition of different EPS fractions are presented in Figure 2e,f. The yield of S-EPS first increased and then decreased with increasing culture pH values. When the pH value was 8.0, the yield of S-EPS reached a maximum of 427.4 mg L^–1^, which was 42.6% higher than that at pH 5.0. However, the yields of LB-EPS and TB-EPS did not change dramatically, and the protein portions of LB-EPS and TB-EPS reached a maximum when the pH was 8.0, accounting for 13.56 and 17.13%, respectively.

The effects of culture temperature on the yield and composition of different EPS fractions are presented in Figure 2g,h. The yields of the three types of EPS first increased and then decreased with increasing culture temperature. The yields of S-EPS, LB-EPS, and TB-EPS reached a maximum at 30 °C, with values of 326.02, 90.87, and 25.58 mg L^–1^, respectively. With increasing culture temperature, the nucleic acid portion of the three types of EPS increased slowly. The protein portions of LB-EPS and TB-EPS reached a maximum at 30 and 27 °C, accounting for 9.72 and 9.34%, respectively.

### 2.3 Adsorption Properties of Different EPS Fractions

**Figure 3:**
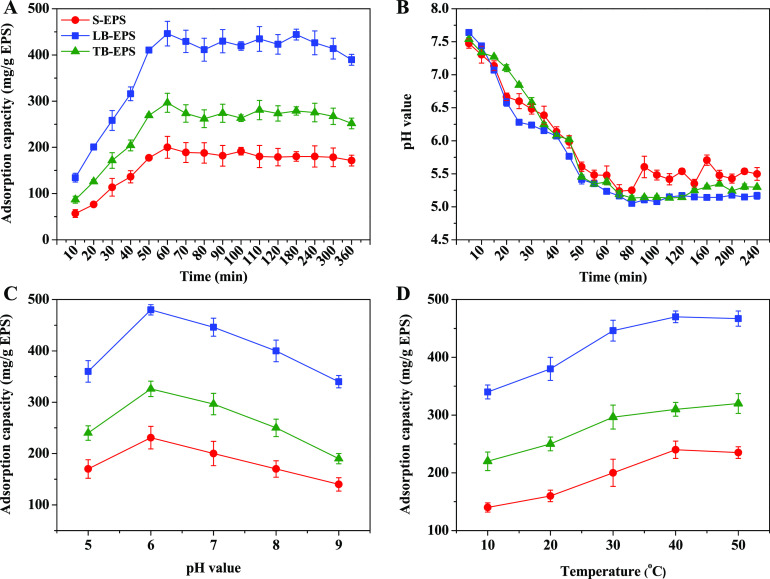
Adsorption capacity for Pb^2+^ by S-EPS, LB-EPS, and TB-EPS in different reaction times (a), pH values (c), and temperatures (d). Variation of pH value for the culture media during the adsorption process of Pb^2+^ (b).

The adsorption capacities of the different EPS fractions and the variations in the pH value of the culture medium are presented in Figure 3a,b. The adsorption process of the three types of EPS proceeded in three stages: fast adsorption followed by slow adsorption and finally adsorption equilibrium. During the 360 min, the fast adsorption stage appeared within the first 30 min, while the slow adsorption stage appeared at 30 to 60 min and the adsorption equilibrium appeared after 60 min. Specifically, throughout the incubation period, the highest Pb^2+^ adsorption capacity was observed for LB-EPS followed by TB-EPS, and the adsorption capacity of S-EPS was the lowest. The Pb^2+^ adsorption capacities of LB-EPS, S-EPS, and TB-EPS reached the highest values of 446.09, 200.06, and 296.48 mg g^–1^ EPS, respectively, at 60 min. The adsorption capacity of LB-EPS was 2.23 and 1.50 times higher than that of S-EPS and TB-EPS, respectively (Figure 3a). As shown in Figure 3b, the pH value of the culture medium decreased during the adsorption reaction. Within 40 min of the reaction, the pH values of the S-EPS, LB-EPS, and TB-EPS reaction systems were reduced by 17.80, 20.54, and 19.50%, respectively, compared with the pH before the reaction. The pH values for the S-EPS, LB-EPS, and TB-EPS reaction systems reached equilibrium at approximately 60 min with values of 5.48, 5.23, and 5.37, respectively.

The effects of pH and temperature on the Pb^2+^ adsorption capacity of the different EPS fractions are presented in Figure 2c,d. At different culture pH values and temperatures, the Pb^2+^ adsorption capacity of LB-EPS was the highest and the adsorption capacity of S-EPS was the lowest. The Pb^2+^ adsorption capacities reached the highest values of 480.03 mg g^–1^ for LB-EPS, 231.54 mg g^–1^ for S-EPS, and 326.33 mg g^–1^ for TB-EPS, respectively, at pH 6.0. In addition, the Pb^2+^ adsorption capacities reached the highest values of 470.32 mg g^–1^ for LB-EPS and 240.61 mg g^–1^ for S-EPS, respectively, at 40 °C. However, the Pb^2+^ adsorption capacity of TB-EPS reached a maximum of 320.14 mg g^–1^ EPS at 50 °C.

### 2.4 Characteristics of Different EPS Fractions before and after Pb^2+^ Adsorption

#### 2.4.1 3D-EEM

**Figure 4:**
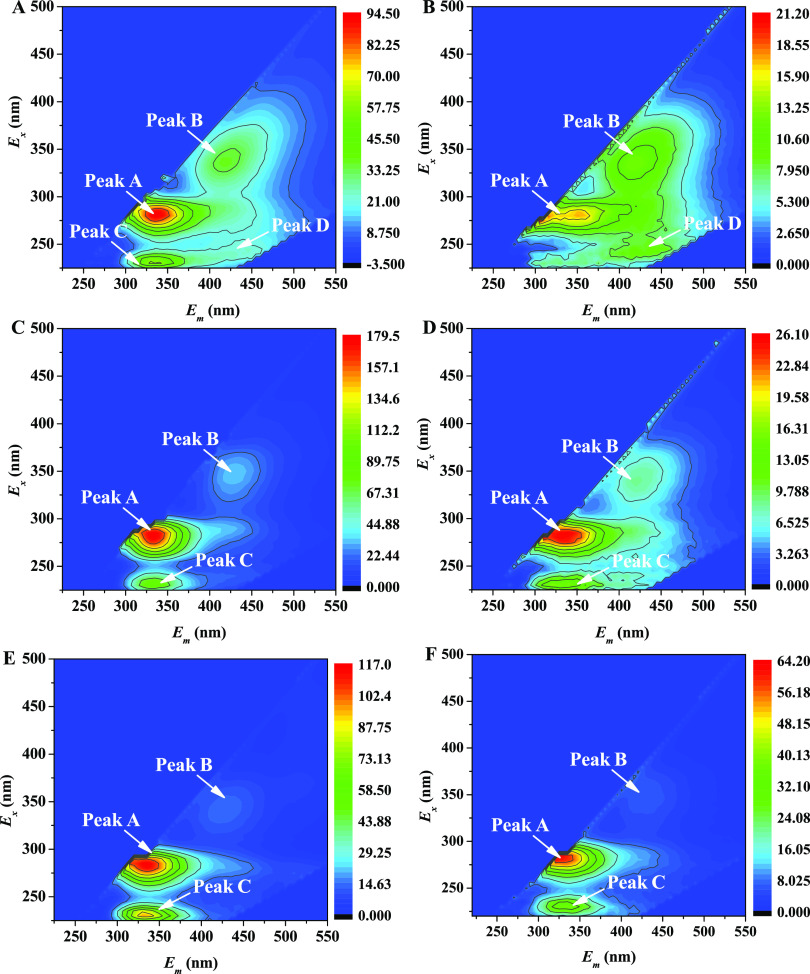
3D-EEM spectra for S-EPS, LB-EPS, and TB-EPS produced by *Enterobacter* sp. FM-1 before and after adsorption of Pb^2+^. (a) S-EPS before adsorption, (b) S-EPS after adsorption, (c) LB-EPS before adsorption, (d) LB-EPS after adsorption, (e) TB-EPS before adsorption, and (f) TB-EPS after adsorption.

The 3D-EEM spectra of three types of EPS are displayed in Figure 4. For S-EPS, four fluorescence peaks were observed before Pb^2+^ adsorption and three fluorescence peaks were observed after Pb^2+^ adsorption (Figure 4a,b). For both LB-EPS and TB-EPS, three fluorescence peaks were observed before and after Pb^2+^ adsorption (Figure 4c–f). The fluorescence spectral positions and fluorescence intensity of each type of EPS before and after Pb^2+^ adsorption are summarized in Table 1.

**Table 1:** 3D-EEM Spectrum Information of S-EPS, LB-EPS, and TB-EPS Produced by *Enterobacter* sp. FM-1 before and after Pb^2+^ Adsorption

	spectral peak information (Ex/Em, intensity)						
	before adsorption			after adsorption			
peak	S-EPS	LB-EPS	TB-EPS	S-EPS	LB-EPS	TB-EPS	existing substances
peak A	280/340, 94.0	285/325, 179.3	285/335, 116.8	285/350, 25.7	285/325, 25.4	285/335, 59.7	tryptophan-like
peak B	345/430, 34.9	345/425, 32.4	345/430, 13.8	335/420, 12.8	345/430, 8.2	345/430, 6.2	humic acid-like
peak C	230/330, 58.6	230/335, 88.1	230/335, 94.8		230/340, 15.1	230/340, 37.7	aromatic protein-like
peak D	245/445, 23.9			245/440, 9.4			fulvic acid-like

The peaks indicated that all EPS types were composed of tryptophan-containing proteins, humic acid-like substances, and aromatic proteins. Peak A located at Ex/Em 280–285/325–350 nm represents a tryptophan-containing fluorescent substance, while peak B located at Ex/Em 335–345/420–430 nm represents a humic acid-like fluorescent substance.^25^ Peak C located at Ex/Em 230/330–340 nm is related to aromatic proteins in EPS.^26^ In addition, S-EPS contained fulvic acids, whose peak was located at Ex/Em 245/440–445 nm.^25^ For LB-EPS, the fluorescence intensity of tryptophan-containing proteins was the strongest; for S-EPS, the fluorescence intensity of humic acids was the strongest; and for TB-EPS, the fluorescence intensity of aromatic proteins was the strongest. Moreover, some significant changes in EPS fluorescence are presented in Figure 4; for instance, after Pb^2+^ adsorption, the fluorescence intensities of humic acid substances in S-EPS, tryptophan-containing proteins in LB-EPS, and aromatic proteins in TB-EPS decreased by 63.3, 85.8, and 60.2%, respectively.

#### 2.4.2 FT-IR

**Figure 5:**
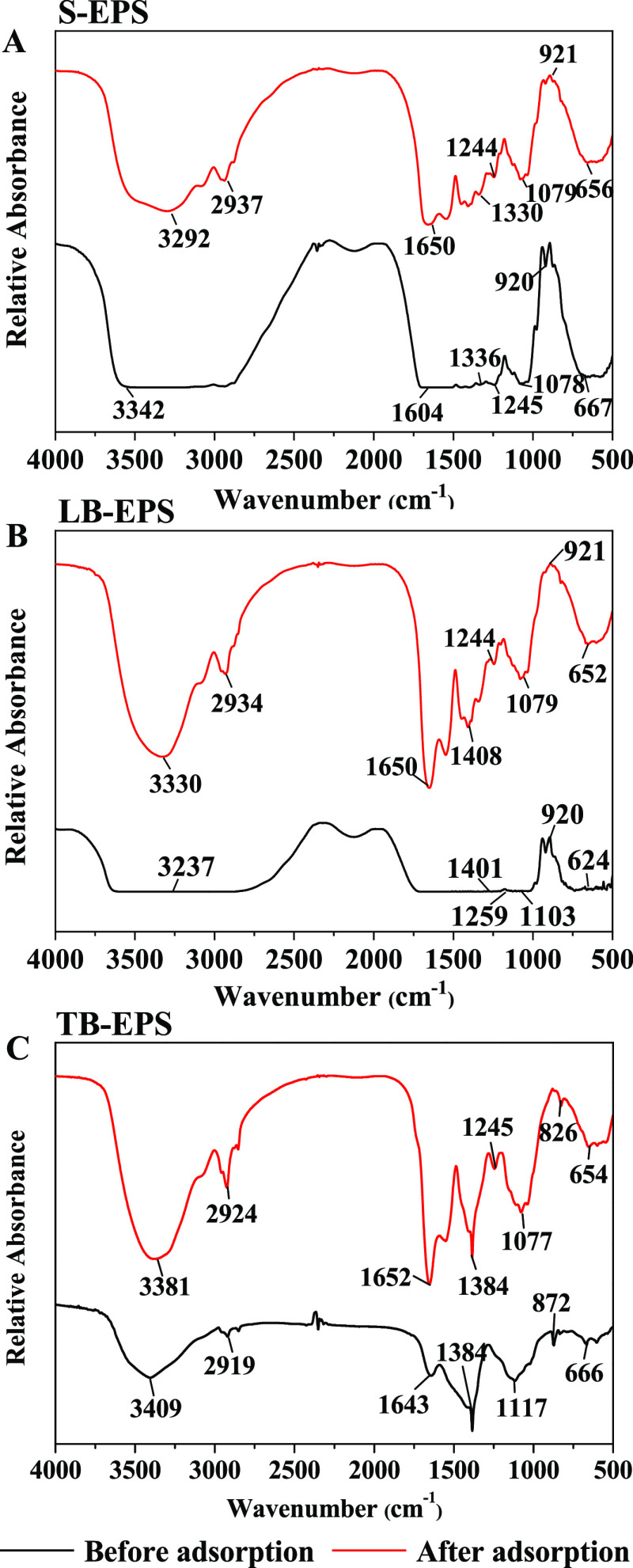
FT-IR spectra for S-EPS (a), LB-EPS (b), and TB-EPS (c) produced by *Enterobacter* sp. FM-1 before and after adsorption of Pb^2+^.

The FT-IR spectra of EPS are displayed in Figure 5 to reveal the functional groups of different EPS fractions produced by FM-1 that were involved in the interaction mechanism. A previous study noted that the adsorption peaks used to characterize the main functional groups of EPS in the range of 1800–600 cm^–1^ are divided into six regions: peaks in the range of 1700–1600 cm^–1^ represent protein amide I, peaks in the range of 1600–1500 cm^–1^ represent protein amide II, peaks in the range of 1500–1300 cm^–1^ represent carboxyl groups and hydrocarbons, peaks in the ranges of 1600–1500 and 1300–1200 cm^–1^ represent protein amide III, peaks in the range of 1200–900 cm^–1^ represent polysaccharides and nucleic acids, and peaks in the range of 900–600 cm^–1^ represent the fingerprint region.^27^

The peak shapes of the three types of EPS were similar before and after adsorption, although the positions of some peaks shifted and the intensity of some peaks increased. The primary bands were observed at peaks ranging from 500 to 1650 cm^–1^ and 2900 to 3500 cm^–1^. The peak at approximately 1650 cm^–1^ indicated the presence of the stretching vibration of C=O in protein amide I, the peak at approximately 1240 cm^–1^ represented N–H in protein amide III, and the peak at 1070 cm^–1^ represented the stretching vibration of C–O–C in polysaccharides.^28^ Some weak absorption peaks were also observed in the fingerprint region, which may belong to phosphate groups found in nucleic acids. Moreover, the wider absorption peak at approximately 3300 cm^–1^ represented the stretching vibrations of N–H and O–H, whereas the peak near 2937 cm^–1^ represented the stretching vibration of C–H, which is the characteristic absorption peak of polysaccharides.^29,30^ Specifically, unlike the other EPS types, the peak observed at 1650 cm^–1^ for LB-EPS before and after adsorption representing the vibration of C=O in protein amide I disappeared, indicating that protein amide I compounds were involved in the adsorption of Pb^2+^ by LB-EPS (Figure 5b). Meanwhile, in the spectra of TB-EPS before and after adsorption, unlike the other EPS types, the peak at 1384 cm^–1^ representing the stretching vibration of C–O in carboxyl groups did not shift, indicating that these carboxyl-containing compounds were not involved in the adsorption of Pb^2+^ by TB-EPS (Figure 5c).

#### 2.4.3 XPS

**Figure 6:**
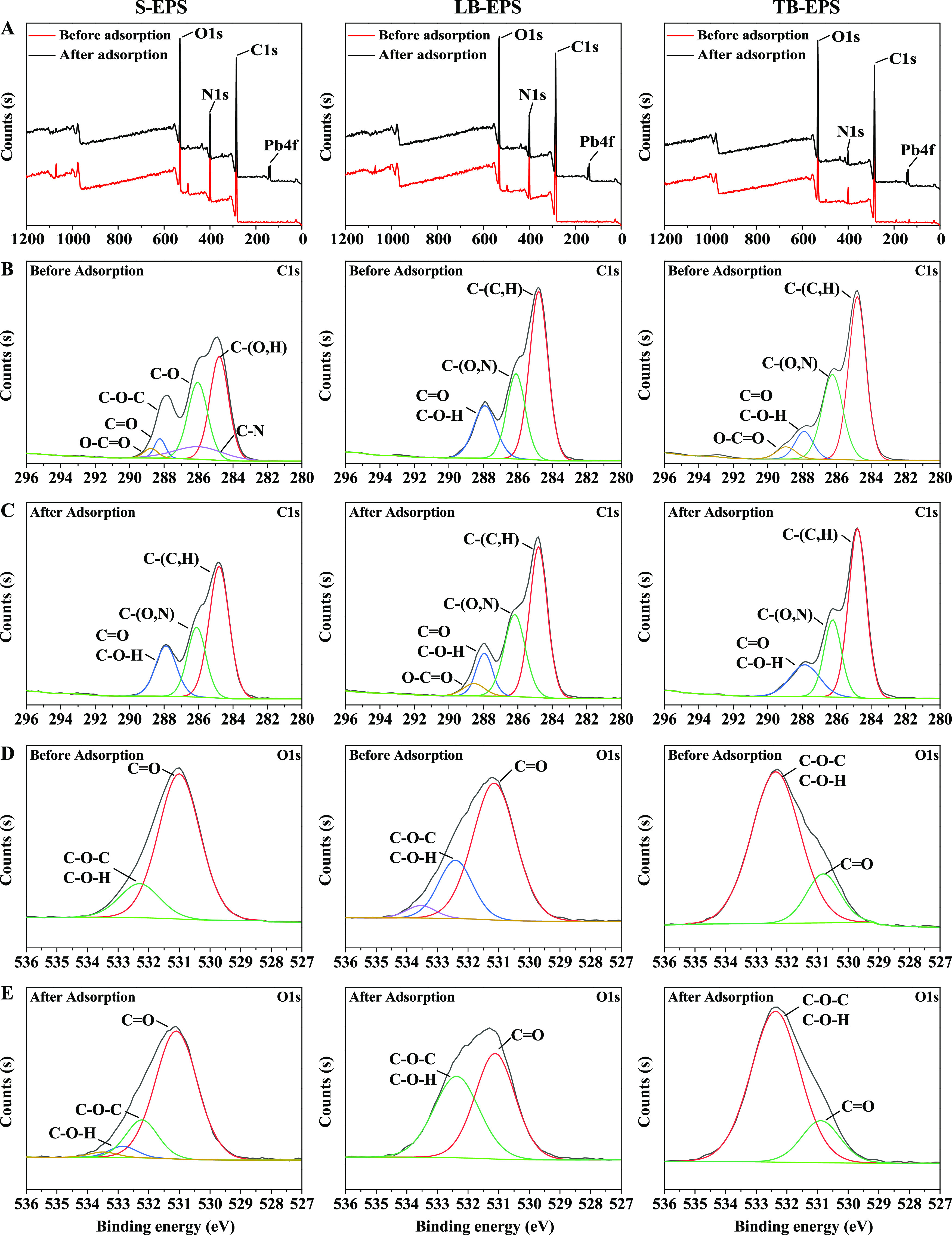
XPS high-resolution spectra of S-EPS, LB-EPS, and TB-EPS produced by *Enterobacter* sp. FM-1 before and after adsorption of Pb^2+^. Full range (a), C1s spectra of EPS before (b) and after (c) adsorption, and O1s spectra of EPS before (d) and after (e) adsorption.

XPS was used to further understand the chemical states and elemental composition of different EPS fractions produced by FM-1. In the current study, high-resolution spectra of the C1s and O1s regions of EPS samples are presented in Figure 6. The full spectra in Figure 6a show that after Pb^2+^ adsorption, a new peak of Pb4f was detected on the EPS surface, indicating that Pb^2+^ was successfully adsorbed on the surface of the three types of EPS. As shown in Figure 6b,c, the C1s peak ranging from 284.7 to 289.5 eV in LB-EPS and TB-EPS was resolved into four different bonds: C–O=O, C–(C–H), C–O, and C=O or C–O–H. The peak at 284.7 eV was associated with C–(C–H) from the side chains of lipids or amino acids and also represented the largest percentage in all three EPS types. In addition, the peak representing C–(C–H) was weaker in S-EPS than in LB-EPS and TB-EPS. However, the positions and proportions of the C1s peaks of each type of EPS did not change significantly before and after Pb^2+^ adsorption. As shown in Figure 6d,e, the O1s peak of all three types of EPS before Pb^2+^ adsorption was resolved into two different bond peaks at 531.3 eV corresponding to C=O and C–O–C in polysaccharides and 533.2 eV corresponding to C–O–H. However, the proportion was different in each type of EPS. For S-EPS and LB-EPS, the percentage of C=O bonds was the largest, with values of 89.69 and 53.41%, respectively. For TB-EPS, the percentage of C–O–C and C–O–H bonds was the largest, with a value of 76.65%. These results indicated significant differences in the composition of different EPS fractions produced by FM-1. Specifically, after Pb^2+^ adsorption, the percentage of the C=O peak in LB-EPS increased significantly by 33.89% (Figure 6e).

## 3 Discussion

A previous study suggested that microorganisms not only have a certain metal tolerance level but also actively adsorb HM ions. Microorganisms also have the ability to achieve HM ion adsorption in a passive manner; the secretion of EPS is particularly relevant during the passive adsorption process.^3^ Adsorption mechanisms between EPS and HMs mainly involve ion exchange, surface complexation, redox reactions, enzymatic reactions, precipitation, etc.^17^ In the present study, the effects of cultivation time, temperature, and pH on the yield and composition of different EPS fractions were investigated. Furthermore, detailed information on EPS before and after Pb^2+^ adsorption was characterized and explored.

Several studies have indicated that changes in cultivation time, pH, and temperature might affect the yield and composition of different EPS fractions produced by bacteria.^31−33^ In the current study, the yield of different EPS fractions produced by FM-1 presented a trend of initially increasing and then decreasing during the process. The yield of different EPS fractions reached a maximum at 24 h, indicating that the stable phase of bacterial growth was conducive to the secretion of EPS; if the cultivation time was too long, the competition among bacteria for survival would increase after entering the endogenous respiration period, which would result in a decrease in the yield of EPS.^26^ Specifically, bacterial metabolism slows during the endogenous respiration period, leading to a reduction in the polysaccharide and protein proportions in EPS components.^34^ In our research, S-EPS secreted by FM-1 reached a maximum at pH = 8.0, although the production of LB-EPS and TB-EPS changed slightly; however, the protein portion of LB-EPS and TB-EPS reached a maximum at pH = 8.0 (Figure 2e,f). With increasing temperature, the yield of EPS and the polysaccharide and protein portions of EPS first increased and then decreased. In addition, EPS secretion by FM-1 reached a maximum at 30 °C and a minimum at 36 °C. The nucleic acid portion of the three types of EPS increased with increasing temperature, indicating that suitable temperature conditions are conducive to the secretion of EPS by FM-1 (Figure 2g,h). Conversely, environmental conditions with low temperature and pH inhibit the activity of enzymes in microbial cells, thereby inhibiting the secretion of EPS.^35^

Throughout cultivation, the proportions of different EPS fractions secreted by FM-1 followed the order of S-EPS > LB-EPS > TB-EPS (Figure 2a), suggesting that S-EPS and LB-EPS are the dominant secreted EPS fractions, consistent with previous studies.^20^ The main components of the three types of EPS are polysaccharides, proteins, and nucleic acids. A previous study noted that bacterial extracellular polysaccharides, proteins, and other substances are negatively charged, and these negatively charged groups play a bridging role in the adsorption of HMs by EPS.^15^ The peaks at 1650, 1240, 1070, and 1070 cm^–1^ in the FT-IR spectra of different EPS fractions further indicated that the main components of EPS are proteins, polysaccharides, and nucleic acids (Figure 5). These substances play an important role in the adsorption of Pb^2+^. Although the main components of the different EPS fractions are the same, the compositions of the EPS fractions are still different. The 3D-EEM analysis showed that S-EPS have a higher content of humic acids, LB-EPS have a higher content of tryptophan-containing proteins, and TB-EPS have a higher content of aromatic proteins (Table 1). These results are similar to the compositions of LB-EPS and TB-EPS secreted by *Aeromonas veronii* strain N8.^19^ Moreover, the XPS analysis also indicated that the composition of different EPS fractions was significantly different.

The Pb^2+^ adsorption capacity of different EPS fractions secreted by FM-1 followed the order of LB-EPS > TB-EPS > S-EPS due to the different binding affinities between different EPS fractions and Pb^2+^. S-EPS are loose substances dissolved in solution; LB-EPS are loosely attached to the cell surface and form a protective film on the cell surface to prevent the toxic effects of HMs on the cell; and TB-EPS tightly bond to the cell surface, making them less easily accessible than LB-EPS.^17,26,36^ During the Pb^2+^ adsorption process, the pH value of the reaction system decreased with increasing adsorption time, indicating that H^+^ was released in the Pb^2+^ adsorption process. Functional groups contained in EPS, such as O–H, C–H, and N–H, have been shown to release H^+^ during adsorption.^37^ According to Ozdemir et al.,^38^ hydroxyl and carboxyl groups are negatively charged and thus attract positively charged cations through electrostatic interactions; furthermore, these groups are involved in metal binding via coordination bonds to form stable complexes in neutral or weakly acidic solutions. Combined with the results presented in the FT-IR analysis (Figure 5), various characteristic peaks of each type of EPS were shifted to varying degrees after Pb^2+^ adsorption, indicating that the active functional groups in EPS include C=O, C–H, O–H, N–H, C–O–C, and phosphate groups that are all involved in the Pb^2+^ adsorption process. Additionally, for LB-EPS, after Pb^2+^ was adsorbed, the vibration of C=O in protein amide I disappeared, indicating that protein amide I compounds were involved in the adsorption of Pb^2+^ by LB-EPS (Figure 5b). Pb^2+^ bound to EPS through ion exchange, and the pH value of the reaction system was the lowest after Pb^2+^ was adsorbed by LB-EPS, which further emphasized that LB-EPS contain more functional groups that can release H^+^, thus enhancing their tolerance and adsorption capacity for Pb^2+^. Moreover, as presented in Figure 3c, the maximum adsorption capacity of different EPS was observed at a pH value of 6.0 but decreased at higher pH values. Huang et al.^10^ reported that the metal binding site of microbes mainly involved the C=O group from the protein fraction under higher environmental stress conditions. The results published by Boyanov et al.^39^ indicated that C=O binding became more pronounced at pH values of approximately 6.4. Furthermore, as presented in the 3D-EEM results, the fluorescence intensity of tryptophan-containing proteins decreased by 85.8% in LB-EPS after Pb^2+^ adsorption (Figure 4d), which is cogent evidence that tryptophan-containing proteins complexed with Pb^2+^ during the adsorption process and their ability to complex with Pb^2+^ was stronger than that of humic acids and aromatic proteins. Based on the results of the 3D-EEM, XPS, and FT-IR analyses, the C=O of protein amide I in tryptophan-containing proteins was mainly responsible for the complexation of LB-EPS and Pb^2+^ in the adsorption process. After complexation with Pb^2+^, the proportion of the C=O peak in the spectrum of LB-EPS increased significantly by 33.89% compared with that before adsorption. Based on this result, the terminal O atom in the C=O of protein amide I played a major role in the complexation process (Figure 6e).

## 4 Conclusions

The yield of different EPS fractions produced by FM-1 reached a maximum when the cultivation time, temperature, and pH were 24 h, 30 °C, and 8.0, respectively. The 3D-EEM spectral analysis indicated that the primary components of S-EPS, LB-EPS, and TB-EPS were humic acids, tryptophan-containing proteins, and aromatic proteins, respectively. During the Pb^2+^ adsorption process, the pH value of the reaction system gradually decreased with increasing reaction time, indicating that H^+^ was released during Pb^2+^ adsorption by EPS. The FT-IR analysis showed that the active functional groups in different EPS fractions included C=O, C–H, O–H, N–H, C–O–C, and phosphoric acid groups, which mainly contributed to the adsorption of Pb^2+^ by ion exchange. Among the three types of EPS, LB-EPS had the greatest ability to interact with Pb^2+^, followed by TB-PES and S-EPS. The pH value of the reaction system was the lowest after Pb^2+^ was adsorbed by LB-EPS, which further emphasized that LB-EPS contain more functional groups that release H^+^. LB-EPS adsorb Pb^2+^ via surface complexation, and the C=O of protein amide I in tryptophan-containing proteins plays an important role in the complexation of LB-EPS with Pb^2+^. However, considering the advantages of microbe-based technology, the future perspectives for further studies include (1) the identification of the specific genes that are associated with the adsorption process in bacterial strains using a metagenomic analysis and (2) the use of molecular techniques to design genetically improved strains with specific metal-binding characteristics. These advances will improve the application of microbial remediation technology to the remediation of more substantial environmental contamination by heavy metals.

## 5 Experimental Section

### 5.1 Bacterial Activation and Stress Cultivation

Strain FM-1 was activated in a liquid Luria–Bertani (LB) medium (containing 10.0 g L^–1^ peptone, 10.0 g L^–1^ NaCl, and 5.0 g L^–1^ yeast extract) and cultivated at 30 °C for 12 h with shaking at 150 rpm. FM-1 bacterial cells were activated and inoculated at a volume of 5% (v/v) in a 250 mL Erlenmeyer flask containing 100 mL of the LB culture medium and various concentrations (0 (control), 100, 200, 300, 400, 600, or 800 mg L^–1^) of Pb^2+^ (prepared with Pb(NO_3_)_2_, analytically pure) to evaluate the Pb^2+^ removal rate and the extracellular and intracellular adsorption capacity of FM-1. The cells were incubated at 30 °C and 150 rpm, and samples were collected after incubation times ranging from 2 to 24 h. After adsorption, the suspension was centrifuged at 8000 rpm for 15 min to collect the supernatant and the cells. The supernatant was filtered through a 0.45 μm microporous membrane, and the residual Pb^2+^ concentration in the supernatant was determined using a flame atomic absorption spectrophotometer (AAnalyst 800, Perkin Elmer, USA). Cells were washed three times with sterile water and then washed several times (20 min each) with 10 mmol L^–1^ EDTA to remove Pb^2+^ on the cell surface; then, the solution was filtered through a 0.45 μm microporous membrane to determine the extracellular Pb^2+^ concentration. Finally, the eluted bacteria cells were digested with microwaves to determine the intracellular Pb^2+^ concentration.^40^ Cell growth was measured by recording the OD_600_ values. The removal rate and adsorption capacity were calculated using formulas 1 and 2, respectively:
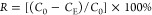
1
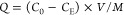
2where *R* (%) is the rate of Pb^2+^ removal, *C*_0_ (mg L^–1^) is the original Pb^2+^ concentration, *C*_E_ (mg L^–1^) is the concentration of residual Pb^2+^ at a certain time point, *Q* (mg g^–1^) is the adsorption capacity of FM-1, *V* (L) is the solution volume, and *M* (g) is the mass of FM-1 after drying at 80 °C to a constant weight.

FM-1 cells cultivated in the LB culture medium (the concentration of Pb^2+^ was 0 and 400 mg L^–1^) for 24 h at 30 °C were harvested by centrifugation at 8000 rpm for 15 min at 4 °C, and cell pellets were prepared using the method described by Naik et al.^41^ Variations in the surface morphology of bacterial cells were observed using a scanning electron microscope (SEM) (Quanta 200 FEG, Oxford Instrument, GB).

### 5.2 EPS Extraction and Determination of the Components

Three additional experiments were performed to explore the effects of different external conditions on EPS production, as described below. (a) The effect of culture time on the secretion of EPS was analyzed by inoculating freshly grown bacterial cultures (5%, v/v) into a liquid LB medium (pH = 7) and culturing them for different times (2–72 h) at 30 °C and 150 rpm. (b) The effect of temperature on the secretion of EPS was analyzed by inoculating freshly grown bacterial cultures (5%, v/v) into a liquid LB medium (pH = 7) and culturing them for 24 h at different temperatures (24–36 °C) and 150 rpm. (c) The effect of pH on EPS secretion was investigated by inoculating freshly grown bacterial cultures (5%, v/v) into a liquid LB medium with different initial pH values (5–9) and culturing them for 24 h at 30 °C and 150 rpm. The pH of the liquid medium was adjusted with 1 mol L^–1^ NaOH or HNO_3_. The initial pH value was determined using a pH meter (A211, Thermo Scientific, USA).

S-EPS, LB-EPS, and TB-EPS were extracted using the method described by Hong et al.^20^ Briefly, after activation, 5% (v/v) of a freshly grown bacterial culture was transferred to LB media and cultivated at 30 °C and 150 rpm for 24 h. For S-EPS extraction, the activated bacterial suspension was centrifuged at 5000 rpm for 10 min at 4 °C, and the supernatant was stored as crude S-EPS. For LB-EPS extraction, the precipitate obtained in the previous step was dissolved in deionized water, ultrasonicated at 40 W for 1 min, and centrifuged at 7000 rpm for 20 min at 4 °C, and the supernatant was stored as crude LB-EPS. For TB-EPS extraction, the precipitate obtained in the previous step was dissolved again in deionized water, and the suspension was heated in a water bath (60 °C, 20 min) and then centrifuged at 10,000 rpm for 20 min at 4 °C; finally, the crude TB-EPS precipitate was collected. The three types of crude EPS were filtered with a polyether sulfone (PES) membrane (0.45 μm) and then transferred to regenerated cellulose (RC) dialysis bags (3500 Da). After 24 h of dialysis at room temperature, pure S-EPS, LB-EPS, and TB-EPS solutions were obtained. The obtained solutions were freeze-dried to a constant weight in a freeze-dryer (FD-1B-50, Bilang, Shanghai, China) at −60 °C and then stored at −20 °C until use.

The protein concentrations of the different types of EPS were determined using the Coomassie Brilliant Blue colorimetric method.^42^ The polysaccharide content was determined by the phenol–sulfuric acid method.^43^ The nucleic acid content was determined by the diphenylamine method.^44^

### 5.3 Analyses of Pb^2+^ Adsorption by Different EPS Fractions

Three additional experiments were conducted to explore the effects of different external conditions on the adsorption capacity of different EPS fractions produced by FM-1, as described below. (a) The effect of reaction time on the adsorption capacity of EPS was analyzed by mixing EPS in a solution containing 100 mg L^–1^ Pb^2+^. Twenty milliliters of the EPS solution was added to a dialysis bag, which was immersed in 100 mg L^–1^ Pb^2+^ and dialyzed at 30 °C, 150 rpm, and pH = 7.0 for different reaction times (10–360 min). During the experiment, samples were removed at intervals of 10–40 min to analyze the change in the equilibrium pH value of the reaction system, which was determined using a pH meter (A211, Thermo Scientific, USA). (b) The effect of temperature on the adsorption capacity of EPS was analyzed by adding 20 mL of the EPS solution to a dialysis bag, which was immersed in 100 mg L^–1^ Pb^2+^ and dialyzed at different temperatures (10–50 °C) at 150 rpm and pH = 7.0. (c) The effect of pH on the adsorption capacity of EPS was investigated by adding 20 mL of the EPS solution to a dialysis bag, which was immersed in 100 mg L^–1^ Pb^2+^ and dialyzed under different initial pH conditions (5–9) at 30 °C and 150 rpm. The pH of the liquid medium was adjusted and measured using the methods described above.

After the reaction, the dialysis bag was stirred in a beaker containing 250 mL of distilled water for 12 h. After filtration with a 0.45 μm microporous membrane, the residual Pb^2+^ concentration in the supernatant was determined using a flame atomic absorption spectrophotometer (AAnalyst 800, Perkin Elmer, USA), and the amount adsorbed was calculated using formula 3:
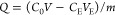
3where *C*_0_ (mg L^–1^) and *C*_E_ (mg L^–1^) are the original Pb^2+^ concentration and Pb^2+^ concentration measured at a specific time point, respectively, *Q* (mg g^–1^ EPS) is the adsorption capacity of EPS, *V* (L) and *V*_E_ (L) are the volume before Pb^2+^ adsorption and volume of the dialysate, respectively, and *m* (g) is the mass of EPS.

### 5.4 EPS Characterization

X-ray photoelectron spectroscopy (XPS), three-dimensional excitation–emission matrix fluorescence spectroscopy (3D-EEM), and Fourier transform infrared spectroscopy (FT-IR) were used to characterize the variations in different EPS fractions and the interactions between Pb^2+^ and EPS.

The main chemical components of EPS before and after the adsorption of Pb^2+^ were analyzed according to their fluorescence characteristic peaks and fluorescence intensity. Samples were placed in a 1 cm quartz colorimeter tube and analyzed using a three-dimensional fluorophotometer (F-7000, Hitachi, Japan). Both the fluorescence spectrum and intensity were recorded at 5 nm increments using excitation wavelengths (Ex) and emission wavelengths (Em) of 200–500 and 280–550 nm, respectively, and a scanning speed of 1200 nm min^–1^.^26^

The FT-IR spectra of EPS before and after the adsorption of Pb^2+^ were analyzed using a Nicolet N10 instrument (Thermo Fisher, USA) to determine the complexation of functional groups and the interactions between Pb^2+^ and EPS functional groups. Freeze-dried EPS and dried and ground anhydrous KBr were milled and mixed in an agate grinder at a mass ratio of 1:100. A KBr powder tablet was used as the background spectrum, the FT-IR wavenumber ranged from 400 to 40,000 cm^–1^ with a resolution of 4 cm^–1^, and scanning was performed in parallel 256 times.^45^ XPS was performed to characterize the changes in the functional groups of EPS before and after Pb^2+^ adsorption using an XPS spectrometer (ESCALAB 250XI, USA). Binding energies were calibrated according to the C1s peak (284.8 eV). XPS spectral peaks were fit using the XPS peak 4.1 soft package.^26^

## Notes

**Yi Li:** Conceptualization, Methodology, Visualization, Writing-original draft, Funding acquisition. **Meifen Xin:** Investigation, Validation, Data curation, Writing-original draft, **Dongyu Xie:** Validation, Data curation, Formal analysis. **Shirui Fan:** Software, Validation, Data curation, Formal analysis. **Jiangming Ma:** Funding acquisition, Supervision. **Kehui Liu:** Funding acquisition, Writing-review & editing. **Fangming Yu:** Writing-review & editing, Supervision, Methodology, Funding acquisition,.
